# Evaluation of commercially available *aroA* delated gene *E. coli O78* vaccine in commercial broiler chickens under Middle East simulating field conditions

**DOI:** 10.1038/s41598-021-81523-x

**Published:** 2021-01-21

**Authors:** Hussein M. Galal, M. I. Abdrabou, Ahmed H. I. Faraag, C. K. Mah, Azza M. Tawfek

**Affiliations:** 1 Department of Microbiology, Faculty of Veterinary Medicine, Cairo University, Giza, Egypt; 2 Department of Cytology and Histology, Faculty of Veterinary Medicine, Cairo University, Giza, Egypt; 3 Botany and Microbiology Department, Faculty of Science, Helwan University, Ain Helwan, Cairo, 11795 Egypt; 4 Outcomes Research Director, APAC & Greater China Clusters, Zoetis Inc., Parsippany, USA; 5 Department of Clinical Pathology, Faculty of Veterinary Medicine, Cairo University, Giza, Egypt

**Keywords:** Bacteria, Vaccines

## Abstract

The broiler industry in the Middle East (ME) faces many challenges related to bacterial infections, including *M. gallisepticum, M. synoviae*, *E. coli,* and other gram-negative bacteria, exacerbated by various errors in the brooding process. Antibiotics use in the first three days of life, such as Linco-Spectin 100 SP, tilmicosin, enrofloxacin, tylosin, colistin, and doxycycline, is the trend in the market to control such challenges. This study aimed to evaluate the efficacy of the newly introduced *aroA E. coli* vaccine (Poulvac *E. coli*) and its ability to reduce over-reliance on the heavy use of antibiotics in the ME. The study was conducted on 160 broiler chicks, divided into eight even groups. Each group was treated differently in terms of antibiotic therapy and ages at the time of Poulvac *E. coli* administration and the challenge of virulent avian pathogenic *E. coli* (APEC), serotype O78. Spray application of Poulvac *E. coli* at seven days of age plus Linco-Spectin 100 SP during the first three days provided the best results for zero mortality after challenge with APEC, while Poulvac *E. coli* at seven days with enrofloxacin during the early three days resulted in 10% mortality. Poulvac *E. coli* hatchery vaccination protected birds against mortality but reduced body weight gain compared to the 7-day group vaccinated with Linco-Spectin 100 SP during the first three days. Poulvac *E. coli* given on day one or day seven did not affect the immune response to concurrent respiratory viral vaccines and, in some cases, improved response. This study shows that Poulvac *E. coli* at seven days of age, together with Linco-Spectin 100 during the first three days, has produced the best results in terms of protection and performance in the ME high presence of avian pathogenic *E. coli* field challenge.

## Introduction

*Escherichia coli *(*E. coli*) is a commensal bacterium in the intestines of chickens. However, some strains, including extra-intestinal avian pathogenic *E. coli* (APEC), may cause invasive infections outside the intestine, namely colibacillosis^1,2^. This disease is responsible for significant economic losses in the poultry industry worldwide due to reduced production, high treatment costs, rejection of carcasses, and mortality^3,4^. APEC exaggerates the severity and complications of Avian Influenza (AI) H9N2, Infectious Bronchitis Virus (IBV) and Velogenic Viscerotropic Newcastle Disease (VVND) endemic to ME and increases post-vaccination reactions associated with spray vaccination, particularly to the Newcastle Disease Virus (NDV) LaSota vaccine and variants of IBV vaccine strains. Antibiotic therapy is used to control this infection; however, there is a significant increase in drug-resistant strains of *E. coli*. Restrictions on the use of antibiotics by the European Union and a reduction in the number of new active ingredients entering the market^5,6^ have been limiting therapeutic effectiveness^7^. Medication for broiler chicks of the first three days of age in ME countries is common in eliminating vertical or hatchery-transmitted bacterial pathogens. Due to the shorter lifespan of the broilers in ME, most producers do not wait for seven days' withdrawal before applying Poulvac *E. coli* (Zoetis), but results appear to be acceptable and better than non-vaccinated. A study in USA^8^ showed that the antibiotics administered in ovo (Gentamicin and Naxcel) were insufficient to interfere with the interaction between the vaccine and the chick's immune system. Therefore, one of the objectives of this study was to assess any effect that common practice might have on the in vivo efficacy of Poulvac *E. coli*. This study aimed to find an alternative to currently used antibiotic therapy for colibacillosis through disease prevention by assessing the effectiveness of commercially available Poulvac *E. coli* vaccine in broiler chickens under ME field conditions. Therefore, the positioning of the vaccine with two heavily used antibiotics in broiler brooding has been explored.

## Material and methods

### Statement

All experiments and methods have been carried out under the relevant guidelines and regulations of the Veterinary Medicine-Cairo University- Institutional Animal Care and Use Committee (Vet. CU. IACUC). Vet. CU. IACUC has approved all experimental protocols, including chicken vaccination and histopathological examination, and relevant protocols. The Code of Ethics of Veterinary Medicine Cairo University-Institutional Animal Care was (Vet CU20022020130). The study was carried out in line with ARRIVE guidelines for the National center of Replacement, Reduction and Refinement of animals in research. (http://www.nc3rs.org.uk/page.asp?id=1357).

### Source of animal

(Ross 308, Aviagen breed) was obtained from a local distributor in Egypt and floor-reared under strict hygienic conditions in previously cleaned and disinfected separated experimental units. Chicks were provided with commercial broiler ration, and ad libitum was provided with water and feed.

### The procedure of animal experiments

Dead birds have been hygienically disposed of by incineration according to the Vet CU IACUC guidelines.

### Experimental chickens

One hundred sixty-one-day-old non-sixed commercial broiler chicks, 40 g body weight were vaccinated at 1 day of age with a coarse spray of Poulvac IB Primer (Zoetis, Olot Manufacturing site, Spain) combined with Poulvac NDW (Zoetis, Campinas Manufacturing site, Brazil) and at 14 days with Nobilis Clone-Ma5 (MSD) to control classical IBV and variant strains and expected NDV challenges. On day one, chicks were administered Egyflu, an inactivated H5N1 reassortant vaccine prepared from strain A/ch/Egypt/A-18-H-09 (produced by the manufacturer Harbin Weike Biotechnology CO., China) for influenza control H5N1 and OL-VAC, an inactivated NDV-vaccine oil emulsion prepared from strain LaSota. (obtained from the manufacturer FATRO CO., Italy) via subcutaneous injection for the control of NDV. All vaccines were administered as directed by the manufacturer.

### Poulvac *E. coli* live attenuated vaccine

Poulvac *E. coli* (Zoetis) is a live AroA gene that has been deleted from the *Escherichia coli* serotype O78 vaccine. The parent strain is virulent *E. coli* O78, isolated in the United Kingdom from the Central Veterinary Laboratories (CVL) in Weybridge in 1995. The initial vaccine lost its plasmid virulence and other storage virulence factors but retained the essential wall structure and metabolic profile of the O78 vaccine. Modification by deleting the *aroA* gene responsible for the biosynthesis of aromatic amino acids attenuated the Poulvac *E. coli* vaccine (Zoetis, Charles City Manufacturing site, USA), which was administered by coarse spray to these different experimental study groups either on day one or at 7 days of age.

### Antibiotics

Two of the most commonly used antibiotics in the broiler industry were utilized and compared in this study, lincomycin-spectinomycin [Linco-Spectin 100 SP (Zoetis, Lot. 17043210A, Suzhou Manufacturing site, China)] and enrofloxacin [Baytril 10% Oral Solution (Bayer)] administered at 225 mg/kg and 10 mg/kg, respectively, during the first three days of drinking water life.

### *E. coli* challenge strain

The *E. coli* O78 strain EC34195 was kindly offered by the Department of Microbiology of the University of Sadat City Faculty of Veterinary Medicine. The challenge was the intra-tracheal inoculation of 100 μl/bird (109 CFU per ml) at 28 days of age in those affected.

### Experimental groups

The 160 experimental chicks were randomized and evenly assigned to different treatment groups, resulting in eight groups of twenty, consisting of two replicates. The design of the experimental study is shown in the following Table 1. Groups A1(LS-7dV-Ch) and A2(LS-7dV-NCh) were administered Linco-spectin 100 for the first three days of life and were vaccinated with Poulvac *E. coli* on day 7. Groups B1(En-7dV-Ch) and B2(En-7dV-NCh) were both administered enrofloxacin for the first three days of life and vaccinated with Poulvac *E. coli* on day 7. Groups C1(1dV-Ch) and C2(1dV-NCh) were both untreated with either antibiotic during the first 3 days of their life and vaccinated with Poulvac *E. coli* the first day.

**Table 1:** Experimental design.

Group no. and description	Chick no	Antibiotic age\day		Poulvac *E. coli* vaccination age\day	*E. coli O78 strain* challenge age\day
		Linco-spectin 100 SP	Enrofloxacin		
A1(LS-7dV-Ch)	20	1st 3 days	–	7	28
A2(LS-7dV-NCh)	20	1st 3 days	–	7	–
B1(En-7dV-Ch)	20	–	1st 3 days	7	28
B(En-7dV-NCh)	20	–	1st 3 days	7	–
C (1dV-Ch)	20	–	–	1	28
C2(1dV-NCh)	20	–	–	1	–
D1(En-Nv-Ch)	20	–	1st 3 days	–	28
D2(En-Nv-NCh)	20	–	1st 3 days	–	–

Both groups D1(En-Nv-Ch) and D2(En-Nv-NCh) were given enrofloxacin for the first 3 days of life and were not vaccinated with Poulvac *E. coli*.

Groups A1 through D1 were challenged by the strain of *E. coli* O78 on day 28, while Groups A2 through D2 was not challenged. Birds in each group were weighed individually at the age of 23, 30, and 35 days.

### Clinical examination, necropsy, and histopathology

Seven days after exposure to *E. coli*, all chicks in the different experimental groups were observed for clinical signs of colibacillosis (ruffled feathers, rales, gasping, nasal discharge, and diarrhea). Dead birds were necropsied, the remaining birds out of 10 birds per treatment group were humanely killed, and post mortem (PM) tests were conducted under the established system^9^. Samples for histopathological examination originating from tracheas and liver of chicks were fixed in 10% neutral—buffered formalin, routinely processed, enclosed in paraffin, and cut and stained haematoxylin and eosin (HE)^10^. Characteristic lesions for colibacillosis infection have been reported, including cloudy air sacs with or without caseous exudate, peritonitis, perihepatitis, and pericarditis^11,12^.

### Blood samples

At 37 days of age, chicken blood was collected from the wing vein or by slaughtering and kept in sloped position at 37 °C for one hour then at 4 °C overnight. Sera were then separated by centrifugation at 3000 rpm/10 min and stored at − 20 °C until testing, which included the detection of H5N1- and NDV-specific antibodies using a hemagglutination inhibition (HI) assay, according to the World Organization of Animal Health (2008)^13^. Several clinical biochemistry parameters were also reported: total serum protein was determined by Biuret's Weichselbaum reaction^14^, bromcresol green was used to determine albumin at 628 nm^15^, blood urea was colorimetrically measured at 578 nm^16^, and creatinine has been calculated using the Jaffe reaction automatic kinetic process^17^.

### Statistical analysis

Statistical analysis was performed using MedCalc Statistical Software 19.5.3. Average body weight and mortality were based on the Chi-square test analysis^18^. However, mean PM lesion was performed based on the Kruskal–Wallis test^19^, the serum biochemical parameters for non-challenged groups and the groups at issue were significantly different across groups.

## Results

### Clinical signs

Clinical signs observed following the *E. coli* challenge included depression, ruffled feathers, and mild diarrhea, with more severe, especially enteric, symptoms seen three days after the challenge. Non-challenged groups remained healthy and asymptomatic.

### Average body weight

The average body weight was evaluated and recorded at 23, 30, and 35 days of age, with lower final weights reached in the groups challenged with *E. coli O78* strain. For 35 days in groups A1, B1, C1 and D1 (*E. coli-*challenged), body weights were 2.14 kg, 2.18 kg, 1.94 kg, and 1.95 kg, respectively, while for groups A2, B2, C2, and D2 (non-challenged), body weights were 2.34 kg, 2.42 kg, 2.4 kg, and 2.1 kg, respectively, as shown in Table 2. Where two-tailed probabilities (P) equal to 0.0323 and the Chi-square normal distribution test showed accepted normality for Chi-square equal to 0.934 and P equal to 0.3339 with Degrees of Freedom (DF) equal to 1 as shown in Table 3.

**Table 2:** Average body weight, mortality, and PM lesion scores.

Group no.		A1*	A2	B1	B2	C1	C2	D1	D2
Average BW age/days	23	–	1.334	–	1.328	–	1.3	–	1.334
	30	1.8	2.14	1.84	2.24	1.74	2.2	1.69	1.88
	35	2.14	2.34	2.18	2.42	1.94	2.4	1.95	2.1
Mortality%		0	0	10	0	0	0	10	0
Average PM lesion score		2.6^ab^	NIL**	2.2^a^	NIL	2.5^ab^	NIL	3^c^	NIL

Means with different letters (a, b) are significantly different at P-value ≤ 0.05.

*A1: LS-7dV-Ch, A2: LS-7dV-NCh, B1: En-7dV-Ch, B2: En-7dV-NCh, C1: 1dV-Ch, C2: 1dV-Ch, D1: En-Nv-Ch, D2: En-Nv-NCh.

**No PM lesions have been observed.

**Table 3:** Average body weight Chi-squared test analysis.

Average body weight age/days	30 days	35 days
Sample size	8	8
Arithmetic mean	1.9413	2.1837
95% CI for the mean	1.7588 to 2.1237	2.0258 to 2.3417
Variance	0.04761	0.03571
Standard deviation	0.2182	0.1890
Standard error of the mean	0.07715	0.06681
F-test for equal variances	P = 0.714	
**T-test (assuming equal variances)**		
Difference	0.2425	
Pooled standard deviation	0.2041	
Standard error	0.1021	
95% CI of difference	0.02361–0.4614	
Test statistic t	2.376	
Degrees of freedom (DF)	14	
Two-tailed probability	P = 0.0323	
**Residuals**		
Chi-squared test for Normal distribution	Accept normality (P = 0.3339) (Chi-squared = 0.934 DF = 1)	

### Mortality and post-mortem lesions

Mortality was observed and recorded for one week after *E. coli* O78 is a challenge. Mortality percentages in groups A1, B1, C1, and D1 (E. coli-challenged) were 0%, 10%, 0%, and 10%, respectively, while mortality remained 0 percent in groups A2, B2, C2, and D4 (non-challenged) as shown in Table 2. Table 4 shows that the result of mortality is not significant as Chi-squared equal to 8 and Degree of Freedom (DF) equal to 7 with P-Value equal to 0.3326.

**Table 4:** Mortality Chi-squared test analysis.

Mortality%	Group no.								
	A1*	A2	B1	B2	C1	C2	D1	D2	
0	1	1	0	1	1	1	0	1	6 (75.0%)
10	0	0	1	0	0	0	1	0	2 (25.0%)
	1 (12.5%)	1 (12.5%)	1 (12.5%)	1 (12.5%)	1 (12.5%)	1 (12.5%)	1 (12.5%)	1 (12.5%)	8
Chi-squared	8								
DF	7								
Significance level	P = 0.3326								
Contingency coefficient	0.707								

PM lesions were measured by cloudiness, turbidity, or deposition of the fibrin membrane to the liver, heart, and air sacs. The average PM lesion score for groups A1, B1, C1, and D1 (*E. coli*-challenged) was 2.6, 2.2, 2.5, and 3, respectively (Table 2). Table 5 showed the nonparametric Kruskal–Wallis test. The p-value equal to 0.391625 greater than 0.05 indicates that the average PM lesion does not provide any reason to conclude that the distributions are different.

**Table 5:** Average PM lesion score (Kruskal–Wallis test).

Average PM lesion	n	Minimum	25th percentile	Median	75th percentile	Maximum	Average Rank	
A1*	1	2.6000	2.600	2.600	2.600	2.600	3.00	
B1	1	2.2000	2.200	2.200	2.200	2.200	1.00	
C1	1	2.5000	2.500	2.500	2.500	2.500	2.00	
D1	1	3.0000	3.000	3.000	3.000	3.000	4.00	
Test statistic	3.0000							
Corrected for ties Ht	3.0000							
Degrees of Freedom (DF)	3							
Significance level	P = 0.391625							

### Serological evaluation of antibody titers for AI H5N1 and NDV by hemagglutination inhibition (HI) assay

Due to limited human resources, ten birds per treatment group have been retained for blood work. The antibody titers for the avian influenza H5N1 virus were evaluated at 21, 27, and 37 days of age and found to be approximately identical between groups. At 37 days, the HI titer (log2) for *E. coli-*challenged groups A1, B1, C1, and D1 was 6, 5.2, 5.7, 5.5, respectively, while the titer for non-challenged groups A2, B2, C2, and D4 was 6.5, 4.5, 5.7, and 6, respectively, as shown in Table 6. The antibody titers shown against the avian influenza H5N1 virus P-value of 0.368 and the Chi-square normal distribution test showed accepted normality for Chi-square equal to 0.277 and P equal 0.5986 with Degrees of Freedom (DF) equal to 1 (Table 7).

**Table 6:** Antibody titers against avian influenza H5N1 and NDV.

Group no		A1^a^	A2	B1	B2	C1	C2	D1	D2
H5N1 age/day	21	3.7		3.7		3.2		4	
	27	5		4.5		3.5		5.7	
	37	6	6.5	5.2	4.5	5.7	5.7	5.5	6
NDV age/day	21	4		3.7		5.2		3.5	
	27	5.2		5.7		5.2		5.5	
	37	6.5	5	6.2	5	5.5	5.7	5.5	5.7

^a^A1: LS-7dV-Ch, A2: LS-7dV-NCh, B1: En-7dV-Ch, B2: En-7dV-NCh, C1: 1dV-Ch, C2: 1dV-Ch, D1: En-Nv-Ch, D2: En-Nv-NCh.

**Table 7:** Chi-square test for antibody titers for H5N1.

Sample	H5N1 27 age/day	H5N1 37 age/day
Sample size	8	8
Arithmetic mean	4.6750	5.6375
95% CI for the mean	3.9590–5.3910	5.1360–6.1390
Variance	0.7336	0.3598
Standard deviation	0.8565	0.5999
Standard error of the mean	0.3028	0.2121
F-test for equal variances		P = 0.368
**T-test (assuming equal variances)**		
Difference		0.9625
Pooled standard deviation		0.7394
Standard error		0.3697
95% CI of difference		0.1696 to 1.7554
Test statistic t		2.604
Degrees of freedom (DF)		14
Two-tailed probability		P = 0.0208
**Residuals**		
Chi-squared test for normal distribution		Accept normality P = 0.5986) (Chi-squared = 0.277 DF = 1)

Antibody titers for NDV virus were evaluated at 21, 27, and 37 days of age. At 37 days, the HI titer (log2) for *E. coli-*challenged groups A1, B1, C1, and D1 was 6.5, 6.2, 5.5, and 5.5, respectively, while the titer for non-challenged groups A2, B2, C2, and D4 was 5, 5, 5.7 and 5.7, respectively, as shown in Table 6. Table 8 showed antibody titers for the NDV virus P-value of 0.042. The chi-square normal distribution test showed accepted normality for Chi-square equal to 0.115 and P-value equal to 0.7349 with Degrees of Freedom (DF) equal to 1.

**Table 8:** Chi-square test for antibody titers for NDV.

Sample	NDV_27_age/day	NDV_37_age/day
Sample size	8	8
Arithmetic mean	5.4000	5.6375
95% CI for the mean	5.2104–5.5896	5.1998–6.0752
Variance	0.05143	0.2741
Standard deviation	0.2268	0.5236
Standard error of the mean	0.08018	0.1851
F-test for equal variances	P = 0.042	
**T-test (assuming equal variances)**		
Difference	0.2375	
Pooled standard deviation	0.4034	
Standard error	0.2017	
95% CI of difference	− 0.1952 to 0.6702	
Test statistic t	1.177	
Degrees of freedom (DF)	14	
Two-tailed probability	P = 0.2587	
**Residuals**		
Chi-squared test for Normal distribution	Accept normality (P = 0.7349) (Chi-squared = 0.115 DF = 1)	

### Histopathological results

#### Trachea

**Figure 1:**
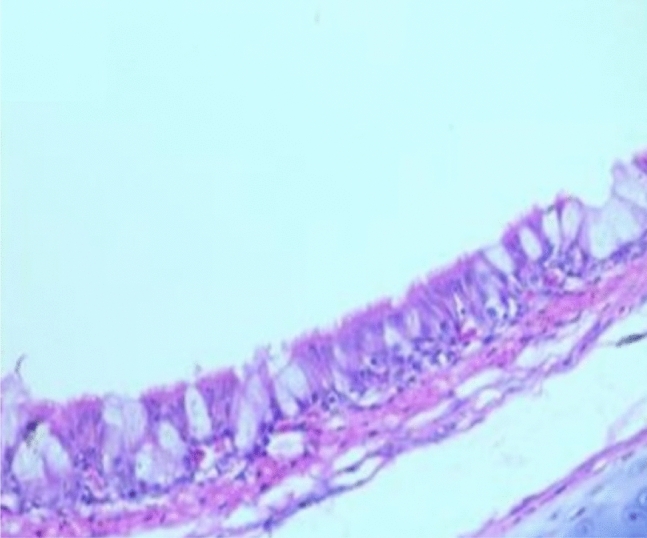
Trachea of unchallenged groups showed normal trachea. (H&E X 400).

**Figure 2:**
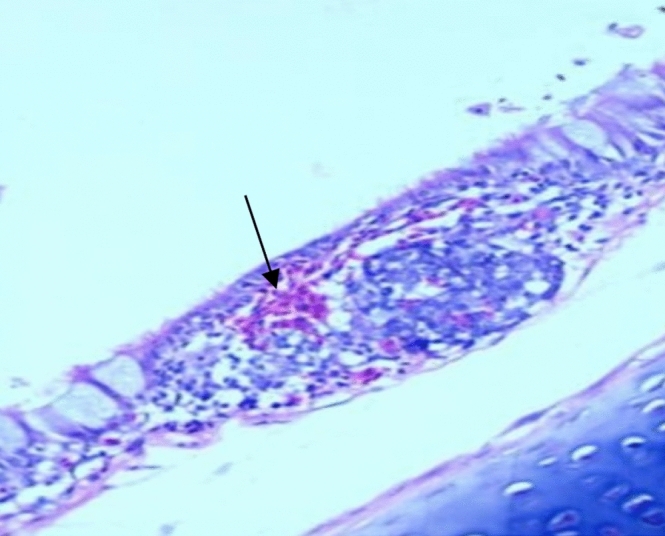
Trachea of Group A1 (LS-7dV-Ch) showed mild thickening in the mucosa, with lymphoid follicle in submucosa extravasated RBCs (H&E X 400).

**Figure 3:**
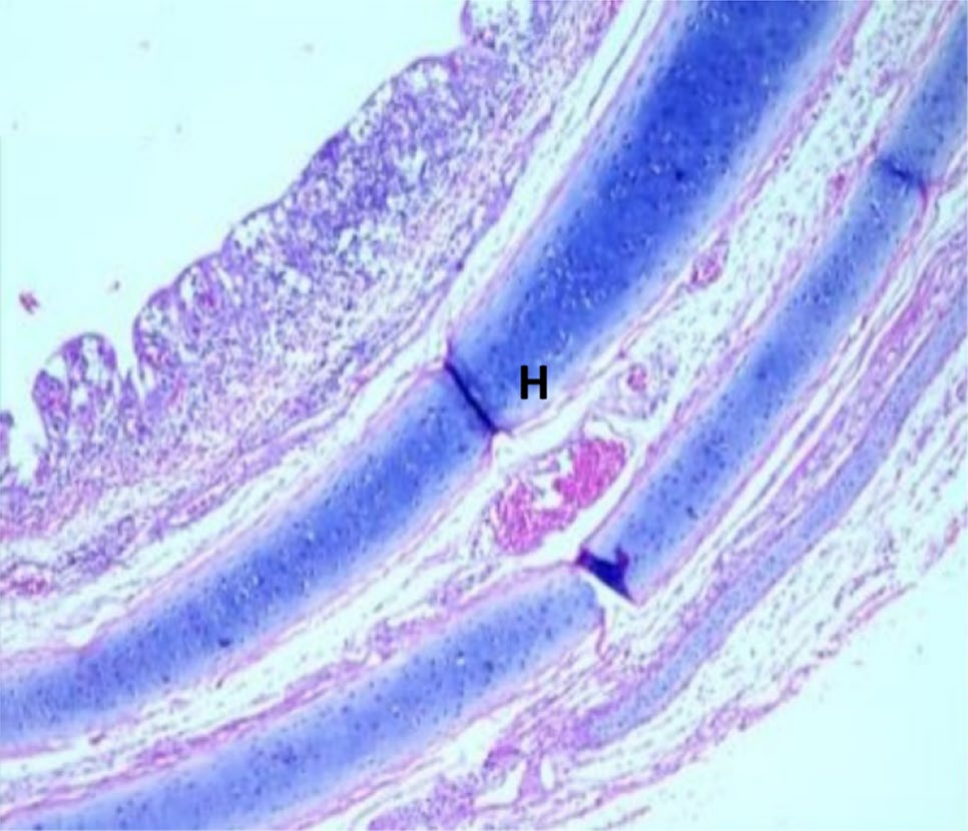
Trachea of Group B1 (En-7dV-Ch) showed mild submucosal edema, hemorrhage, and congestion with loss of columnar epithelium replaced by a single layer of regenerative cells (H&E X 100).

**Figure 4:**
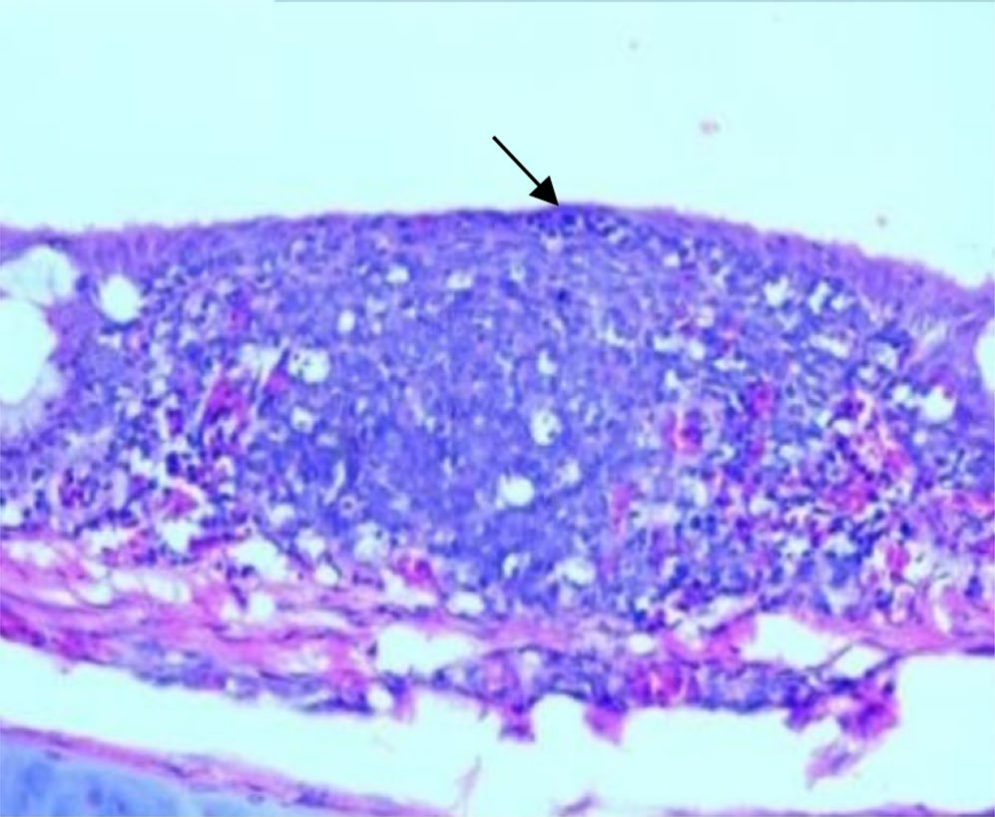
Trachea of Group C1 (1dV-Ch) showed mild submucosal edema, hemorrhage, and congestion with loss of columnar epithelium replaced by a single layer of regenerative cells (H&E X 100).

Histopathological examination revealed that between the different unchallenged groups. Figure 1 shows a normal trachea with a normal mucosal layer formed by pseudostratified ciliated columnar epithelium with goblet cells and submucosa formed by dense fibrous tissue. Figure 2: Group A1 (LS-7dV-Ch) shows mild focal thickening of the epithelium with submucosal lymphocytes nodules (+ 1); meanwhile, the severity of histological changes ranged from moderate {Groups B1 (En-7dV-Ch) & C1 (1dV-Ch)} to severe {Group D1 (En-Nv-Ch)} + 2 and + 3 with focal hyperplasia of tracheal epithelium and focal absence of columnar epithelium replaced by a single layer of regenerative cells. Submucosal infiltration with lymphocytic nodules, accompanied by moderate to severe submucosal congestion and hemorrhage (Figs. 3, 4). Table 9 showed the severity index of histopathological changes in the broiler chicken trachea in all groups.

**Table 9:** Results of histopathological score lesions of the trachea of broiler chickens in a different group with severity index.

Groups	Congestion	Hemorrhage	Thickening of Mucosa	Hyperplasia of lining epithelium	Inflammatory cells	Epithelial degeneration	Mucous glands
A1^a^	**+**	**+**	**+**	**+**	**+**	**+**	−
A2	−	−	−	−	−	−	−
B1	**++**	**+**	**++**	**++**	**++**	**+**	−
B2	−	−	−	−	−	−	−
C1	**+**	**+**	**++**	**+**	**+**	**+**	**+**
C2	−	−	**+**	**+**	**+**	−	−
D1	**+++**	**+++**	**++**	**+++**	**+++**	**++**	**++**
D2	−	−	−	−	−	−	−

−: normal +: Mild lesions ++: Moderate lesion +++ : Severe lesion.

^a^A1: LS-7dV-Ch, A2: LS-7dV-NCh, B1: En-7dV-Ch, B2: En-7dV-NCh, C1: 1dV-Ch, C2: 1dV-Ch, D1: En-Nv-Ch, D2: En-Nv-NCh.

#### Liver

**Figure 5:**
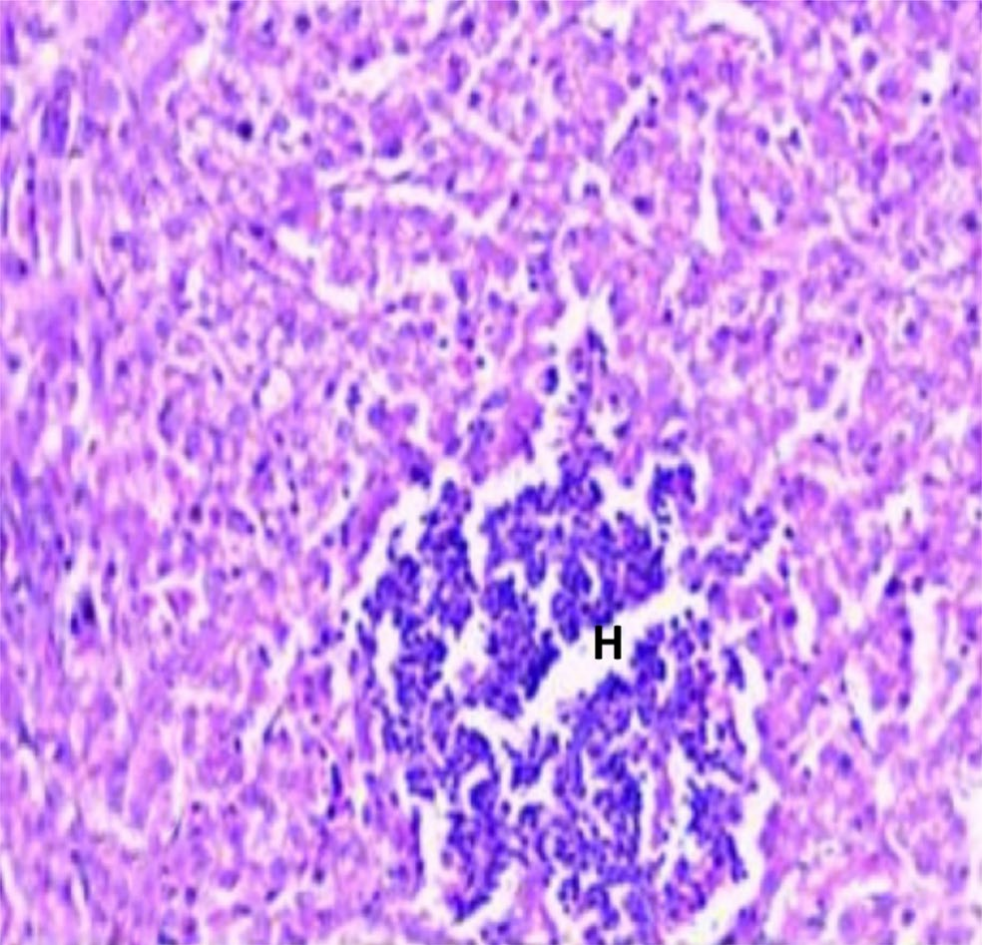
Liver of group A1 (LS-7dV-Ch) showed focal aggregation of histiocytes (H&E X 400).

**Figure 6:**
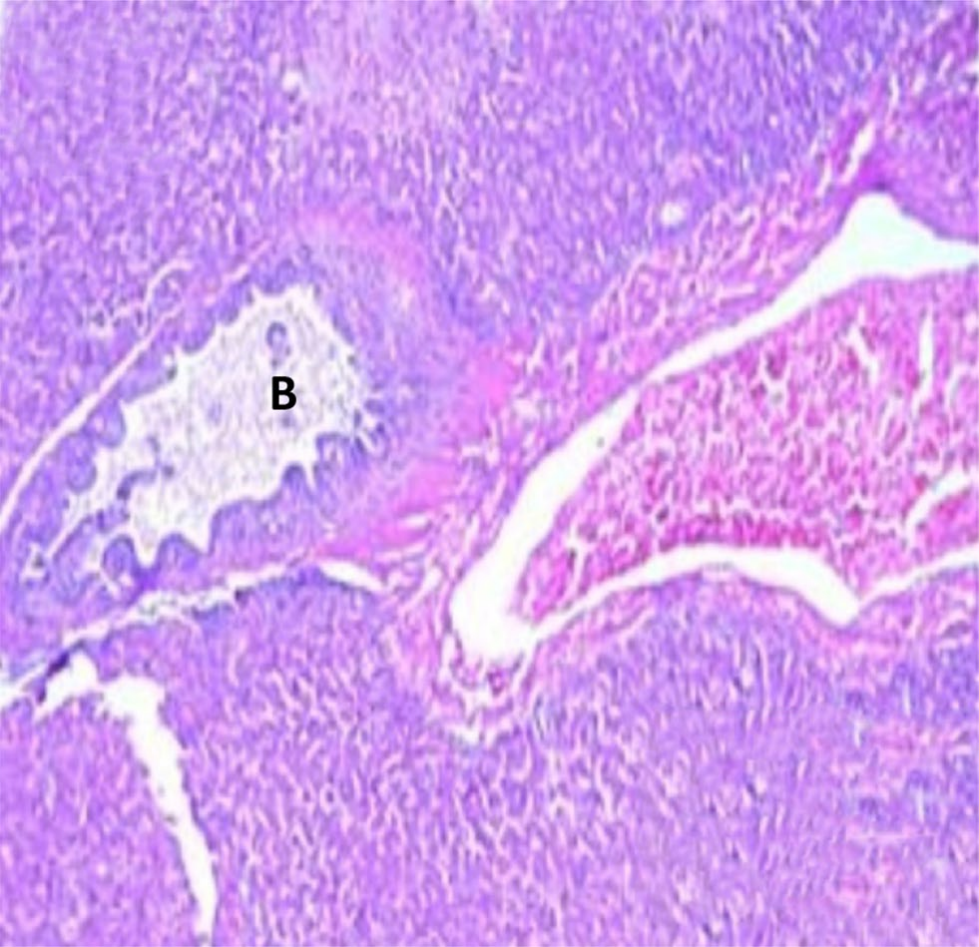
The liver of group C1 (1dV-Ch) showed congested blood vessels with bile ductules' activation (H&E X 100).

**Figure 7:**
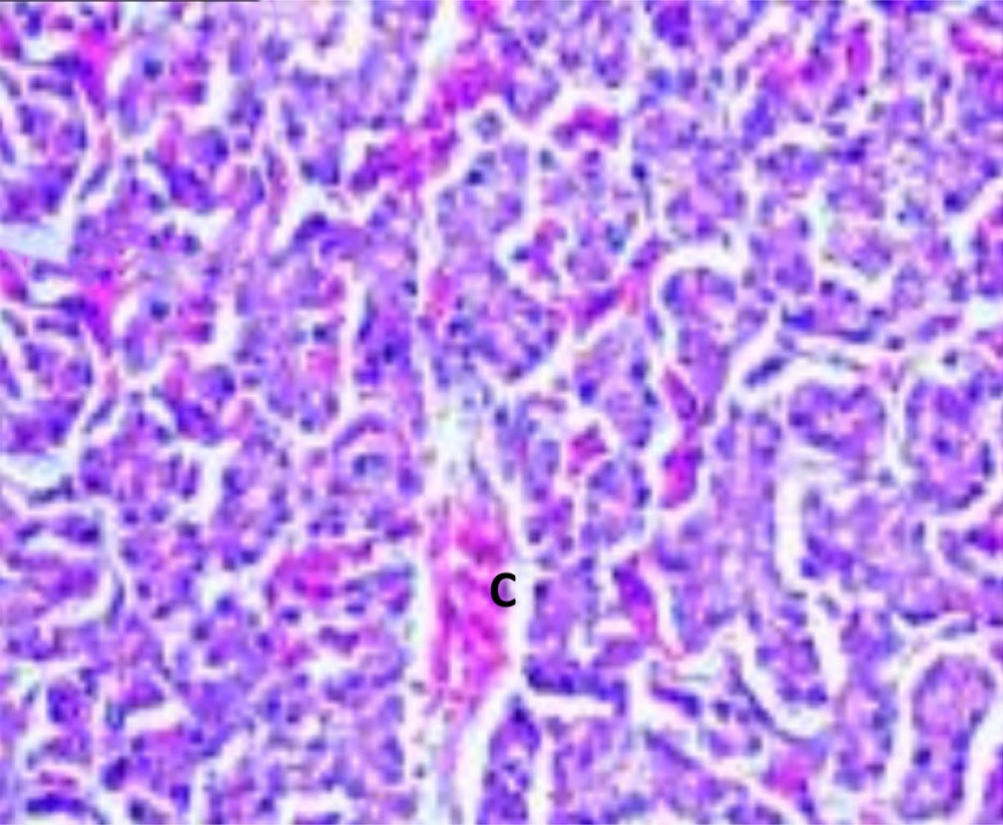
The liver of group B1 (En-7dV-Ch) showed congested sinusoids (H&E X 400).

Histopathological changes in different groups showed mild congestion of the blood vessels, and hepatocytes exhibited mild to severe vacuolar degeneration. Group A1 (LS-7dV-Ch) offers a focal aggregation of histiocytes (Fig. 5), while groups B1 (En-7dV-Ch) & C1 (1dV-Ch) exhibit congestion in the blood sinusoid with thrombus formation and bile ductule proliferation (Figs. 6, 7). Group D1 (En-Nv-Ch) showed severe congestion with prominent thrombus formation and inflammatory cell aggregation. The severity index for each histopathological change in the liver of broiler chickens in all groups has been shown in Table 10.

**Table 10:** Results of histopathological score lesions of the liver of broiler chicken for different groups with severity index.

Group no	Congested B.V	Hemorrhage	Thrombus	Vacuolar degeneration	Necrosis	Inflammatory cells	Hyperplasia of bile ductules
A1^a^	**+**	**−**	**−**	**+**	**−**	**+**	**+**
A2	**+**	**−**	**+**	**−**	**−**	**−**	**−**
B1	**++**	**−**	**++**	**+**	**−**	**−**	**+**
B2	**+**	**+**	**+**	**+**	**−**	**−**	**−**
C1	**++**	**−**	**++**	**−**	**−**	**+**	**+**
C2	**+**	**−**	**+**	**+**	**−**	**+**	**++**
D1	**+++**	**++**	**++**	**+**	**−**	**++**	**++**
D2	**+**	**−**	**−**	**++**	**−**	**++**	**−**

^a^A1: LS-7dV-Ch, A2: LS-7dV-NCh, B1: En-7dV-Ch, B2: En-7dV-NCh, C1: 1dV-Ch, C2: 1dV-Ch, D1: En-Nv-Ch, D2: En-Nv-NCh.

### Results of serum biochemical parameters

Statistical analyses of the results of the total serum protein, albumin, urea, and creatinine *E. coli*-challenged and non-challenged groups are shown below in Tables 11, 12;

**Table 11:** Results of serum biochemical parameters for non-challenged groups.

	TP	Albumin	Urea	Creatinine
A2^a^	5.53 ± 0.21 a	1.70 ± 0.10 a	3.90 ± 0.10 a	0.68 ± 0.03 a
B2	5.23 ± 0.31 a	1.97 ± 0.21 a	4.45 ± 2.55 a	0.70 ± 0.10 a
C2	4.07 ± 1.15 b	1.97 ± 0.15 a	5.30 ± 2.30 a	0.87 ± 0.73 a
D2	4.00 ± 0.60 b	1.85 ± 0.15 a	5.10 ± 2.74 a	0.62 ± 0.08 a

The same letters within the same column are not significantly different; however, other letters indicate significant differences between treatments (Duncan test, P ≤ 0.05).

^a^A2: LS-7dV-NCh, B2: En-7dV-NCh, C2: 1dV-Ch, D2: En-Nv-NCh.

**Table 12:** Results of serum biochemical parameters for challenged groups.

	TP	Albumin	Urea	Creatinine
A1^a^	3.65 ± 0.45 a	1.60 ± 0.10 a	3.73 ± 1.46 b	0.40 ± 0.10 b
B1	3.58 ± 0.18 ab	1.75 ± 0.25 a	6.10 ± 0.40 ab	0.88 ± 0.30 a
C1	3.55 ± 0.25 ab	1.50 ± 0.10 a	3.40 ± 0.40 b	0.65 ± 0.02 ab
D1	3.45 ± 0.05 b	1.65 ± 0.45 a	11.50 ± 4.01 a	0.90 ± 0.30 a

Means with different letters (a, b) within the same column are significantly different (Duncan test, P ≤ 0.05).

^a^A1: LS-7dV-Ch, B1: En-7dV-Ch, C1: 1dV-Ch, D1: En-Nv-Ch.

Statistical analyses of the results of serum biochemistry revealed that mean values of total serum proteins, albumin, urea, and creatinine showed non-significant changes between non-challenged groups (A2, B2, C2, and D2) with only significant differences in total protein between samples collected from Groups A2& B2 and those collected from Groups C2& D2.

Statistical analysis of total protein, urea, and creatinine results revealed significant differences between different *E. coli* challenged groups; however, there were no significant differences between different groups for total albumin. In other words, the A1 group showed a significant increase in total protein compared to group D1. Meanwhile, groups B1 & D1 showed a significant increase in urea and creatinine than groups A1& C1.

## Discussion

*E. coli* are ubiquitous bacteria found in all poultry intestines, and most strains may be considered non-pathogenic, part of the microbiome-related bacterial community*.*

However, some strains, including extra-intestinal avian pathogenic *E. coli* (APEC), can cause invasive infections outside the intestine, namely colibacillosis^1,2^, and are reported to cause significant economic losses to the poultry industry^20^. ME Poulvac *E coli* sprays application of day-old broilers has been reported to help reduce the mortality associated with co-infection by IBV variant 02 and APEC from 25% in the control group 10%^21^. However, under ME field conditions and due to the low quality of day-old chicks, broiler chick medicines are common in the first three days of age to eliminate vertical or hatchery-transmitted bacterial pathogens.

Simultaneously, in vitro assays have already shown that Poulvac *E. coli* is sensitive to Linco-Spectin 100 SP and Baytril 10%, commonly used in ME. Our objective was to measure any effect that common practice might have on the efficacy (in vivo) of Poulvac *E. coli* to best position the vaccine with two heavily used broiler-brooded antibiotics.

In this study, 160 1-day commercial broilers were reared under strict hygienic conditions, divided into eight groups of 20 chicks each (A1, A2, B1, B2, C1, C2, D1, and D2).

Chicks in experimental groups A1 (LS-7dV-Ch) and A2 (LS-7dV-NCh) were both administered Linco-Spectin 100 during the first three days of life and vaccinated with Poulvac *E. coli* on day 7.

Chicks in groups B1 (En-7dV-Ch) & B2 (En-7dV-NCh) were both given enrofloxacin for the first 3 days of life and vaccinated with Poulvac *E. coli* on day 7. Chicks in groups C1 (1dV-Ch) & C2 (1dV-NCh) were vaccinated with Poulvac *E. coli* on 1 day of age. Finally, chicks in groups D1 (En-Nv-Ch) and D2 (En-Nv-NCh) were administered enrofloxacin for the first three days of their life. Chicks in groups A1, B1, C1, and D1, were exposed to the virulent strain of *E. coli* O78 on day 28. All chicks in the different experimental groups were observed for clinical signs of colibacillosis, mortality, PM lesions, histopathological changes, serological evaluation of AIV H5N1 and NDV, and clinicopathological changes.

The results of this study showed that all vaccine treatments had significant reductions in the incidence and severity of airsacculitis-by far the most common lesion (groups A1, B1, and C1). However, there was a difference in *E. coli* protection between the different groups based on prior antibiotic prophylaxis. Out of the four groups in a challenge, birds in groups A1 (LS-7dV-Ch) and C1 (1dV-Ch) showed 100% protection against mortality, while birds in groups B1 (En-7dV-Ch) and D1 (En-Nv-Ch) showed 10% mortality. The efficacy of Poulvac *E. coli* given daily by coarse spray was not compromised by prior gentamicin or Naxcel injection, either in ovo or subQ. This suggests that these antibiotics' levels were insufficient to interfere with the interaction between the vaccine and the chick's immune system daily^8^.

In group C1 (1dV-Ch), despite birds' protection against mortality, the average body weights at day 35 were numerically lower than birds of group A1 (LS-7dV-Ch). For non-challenged groups, all vaccine treatments [groups A2 (LS-7dV-NCh), B2 (En-7dV-NCh), and C2 (1dV-NCh)] showed a higher average body weight at 35 days than non-vaccinated group D2 (En-Nv-NCh). The observed results were similar to previous study and to the recent field trial in Saudi Arabia where Linco-Spectin 100/Poulvac *E. coli* treated flocks showed superior average live body weight, dressed weight, FCR and EPI compared to their current antibiotics only treated flocks with 58 g, 47 g, 5 points and 10.53 points of differences, respectively^22,23^.

The effect of Poulvac *E. coli* on the immune response to concurrent AI H5 and NDV vaccinations was evaluated by the detection of antibody titers at different ages against each particular agent by the HI test. Results have shown that the use of Poulvac *E. coli* on day-old or day 7 with prior antibiotic prophylaxis in the first three days does not affect the immune response to these vaccines. Also, there was an improvement in both viral agents' immune responses, particularly in group A1 (LS-7dV-Ch)^21,24^.

The histopathological examination results showed a normal histological pattern of tracheas collected from birds of non-challenged groups with mild histopathological changes in the liver from the same birds. While the histopathological severity index varied between the different challenged groups, birds of group A1(LS-7dV-Ch) showed the mildest tracheal (mild focal thickening of the epithelium with submucosal lymphocytes nodules + 1) and liver lesions (focal aggregation of histiocytes in the liver), followed by birds in groups B1(En-7dV-Ch) & C1(1dV-Ch) (moderate lesions) and D1(En-Nv-Ch) (severe lesions), respectively.

These results suggest that treatments with the Poulvac *E. coli* vaccine significantly reduced the severity of lesions in both the trachea and liver compared to the non-vaccinated group D1(En-Nv-Ch) with a higher level of protection in group A1 (LS-7dV-Ch)^21,25^. The serum biochemical parameters were aligned with clinical signs, mortality, necropsy, and histopathology findings where group A1(LS-7dV-Ch) showed significant differences in total protein, urea, and creatinine compared to group D1 (En-Nv-Ch). Higher total protein, combined with a lack of difference in total albumin, indicates higher globulin levels in group A1 (LS-7dV-Ch) birds. Meanwhile, urea and creatinine levels in groups A1(LS-7dV-Ch) and C1(1dV-Ch) were significantly lower than in groups B1(En-7dV-Ch) and D1(En-Nv-Ch).

## Conclusion

These results conclude the efficacy of Poulvac *E. coli* given at seven days old by coarse spray was not compromised by prior Linco-Spectin 100 medication at 1st 3 days in commercial broilers. On the other hand, the enrofloxacin level slightly interfered with this vaccine's interaction and the chick's immune system. The protection may not have been as complete as reflected in the 10% mortality. This study proves that Poulvac *E. coli* at seven days of age, together with Linco-Spectin 100 during the first 3 days, gave the best results in terms of protection and performance in the ME that has a high presence of avian pathogenic *E. coli* field challenge.

## Abbreviations

AI: Avian influenza
APEC: Avian pathogenic *E. coli*
CVL: Central Veterinary Laboratories
EPI: European performance index
FCR: Feed conversion ratio
HE: Haematoxylin and eosin
IBV: Infectious bronchitis virus
ME: Middle East
NDV: Newcastle disease virus
PM: Post-mortem
SP: Soluble powder
Vet. CU. IACUC: Veterinary Medicine Cairo University-Institutional Animal Care and Use Committee
VVND: Velogenic viscerotropic Newcastle disease
