# Author Correction: Evaluation of commercially available *aroA* delated gene *E. coli O78* vaccine in commercial broiler chickens under Middle East simulating field conditions

**DOI:** 10.1038/s41598-021-94415-x

**Published:** 2021-07-21

**Authors:** Hussein M. Galal, M. I. Abdrabou, Ahmed H. I. Faraag, C. K. Mah, Azza M. Tawfek

**Affiliations:** 1 Department of Microbiology, Faculty of Veterinary Medicine, Cairo University, Giza, Egypt; 2 Department of Cytology and Histology, Faculty of Veterinary Medicine, Cairo University, Giza, Egypt; 3 Botany and Microbiology Department, Faculty of Science, Helwan University, Ain Helwan, Cairo, 11795 Egypt; 4 Outcomes Research Director, APAC & Greater China Clusters, Zoetis Inc., Parsippany, USA; 5 Department of Clinical Pathology, Faculty of Veterinary Medicine, Cairo University, Giza, Egypt

Correction to: *Scientific Reports* https://doi.org/10.1038/s41598-021-81523-x, published online 21 January 2021

The original version of this Article contained errors.

In the Introduction section,

“Due to the shorter lifespan of the broilers in ME, most producers do not wait for seven days' withdrawal before applying Poulvac *E. coli* (Zoetis), but results appear to be acceptable and better than non-vaccinated. Due to the shorter lifespan of the broilers in ME, most producers do not wait for seven days of withdrawal of Poulvac *E. coli* (Zoetis), but the results appear to be good and better non-vaccinated broilers.”

now reads:

“Due to the shorter lifespan of the broilers in ME, most producers do not wait for seven days' withdrawal before applying Poulvac *E. coli* (Zoetis), but results appear to be acceptable and better than non-vaccinated.”

In the Discussion section,

“The histopathological examination results showed a normal histological pattern of tracheas collected from birds of non-challenged groups with mild histopathological changes in the liver from the same birds. While the histopathological severity index varied between the different challenged groups, birds of group A1(LS-7dV-Ch) showed the mildest tracheal (mild focal thickening of the epithelium with submucosal lymphocytes nodules + 1) and liver lesions (focal aggregation of histiocytes in the liver), followed by birds in groups B1(En-7dV-Ch) & C1(1dV-Ch) (moderate lesions) and D1(En-Nv-Ch) (severe lesions), respectively.

The histopathological examination results showed a normal histological pattern of trachea collected from non-challenged groups of birds with mild histopathological changes in the liver from the same birds. While the histopathological severity index varied from one group to another, group A1(LS-7dV-Ch) birds showed the mildest tracheal (mild focal thickening of the epithelium with submucosal lymphocytes nodules + 1) and liver lesions (focal aggregation of histiocytes in the liver) followed by birds in groups B1(En-7dV-Ch) &C1(1dV-Ch) (moderate lesions) and D1(En-Nv-Ch) (severe lesions).”

now reads:

“The histopathological examination results showed a normal histological pattern of tracheas collected from birds of non-challenged groups with mild histopathological changes in the liver from the same birds. While the histopathological severity index varied between the different challenged groups, birds of group A1(LS-7dV-Ch) showed the mildest tracheal (mild focal thickening of the epithelium with submucosal lymphocytes nodules + 1) and liver lesions (focal aggregation of histiocytes in the liver), followed by birds in groups B1(En-7dV-Ch) & C1(1dV-Ch) (moderate lesions) and D1(En-Nv-Ch) (severe lesions), respectively.”

The original Article has been corrected.

